# Coinfection rates of avian blood parasites increase with latitude in parapatric host species

**DOI:** 10.1017/S0031182022001792

**Published:** 2023-04

**Authors:** Naima C. Starkloff, Spencer C. Galen

**Affiliations:** 1 Department of Biology, Emory University, Atlanta, GA 30322, USA; 2 Department of Biological Sciences, University at Albany, State University of New York, Albany, NY 12222, USA; 3 New York State Museum, Albany, NY 12230, USA; 4 Biology Department, Loyola Science Center, University of Scranton, Scranton, PA 18510, USA

**Keywords:** *Catharus*, coinfection, Haemosporida, latitudinal diversity gradient, *Leucocytozoon*

## Abstract

Animals are frequently coinfected with multiple parasites concurrently, and advances in our sampling of these complex intra-host parasite communities have revealed important ecological impacts on their hosts. However, the spatial distributions and environmental determinants of parasite coinfection remain infrequently studied. Here, we investigated the drivers of haemosporidian blood parasite coinfection in the Bicknell's thrush (*Catharus bicknelli*) and grey-cheeked thrush (*Catharus minimus*), parapatric sister species that occur across a broad latitudinal range in northern North America. Using 298 samples from across the distributions of these species, we found high overall infection (86%) and coinfection (41%) rates within host populations. Coinfection rates within populations were highly variable across sampling sites, ranging from 7 to 75%. Latitude was a more important predictor of coinfection frequency than host species identity, with coinfections becoming more abundant at higher latitudes. The 2 host species exhibited similar parasite faunas, and an analysis of the co-occurrence patterns among haemosporidians showed that host species identity was largely not a factor in structuring which parasites were found within coinfections. To our knowledge, this is the first study to illustrate a reverse latitudinal gradient in coinfection frequency in a eukaryotic parasite system. Further work is necessary to determine whether vector ecology or some other factor is the primary proximate driver of this pattern.

## Graphical abstract

**Figure:**
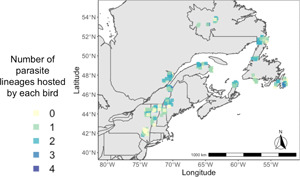

## Introduction

The simultaneous infection of a host individual with multiple parasites is a widespread phenomenon in ecological communities. These coinfections may have far-reaching consequences for our understanding of the ecology and evolution of infectious diseases (Vaumourin *et al*., 2015), and data from natural and experimental infections indicate that coinfections may have different consequences for host fitness than single infections. These effects range from reduced growth rates (Marzal *et al*., 2013) and decreased survival probability (Pigeault *et al*., 2018) observed in natural populations, to elevated short-term virulence caused by coinfections over short time frames in experimental systems (Palinauskas *et al*., 2011; Tang *et al*., 2020). Alternatively, potential antagonism between simultaneously infecting parasites can decrease parasite virulence in hosts. For example, Degarege *et al*. (2012) demonstrated that human patients co-infected with at least 1 species of helminth had less severe *Plasmodium falciparum* infections than patients not infected by helminths. Similarly, while increased concentrations of glucorticoid hormones can increase the cost of parasite infection, it has been shown that coinfection with multiple parasites can reduce this negative effect (Schoenle *et al*., 2019).

Given that coinfections impact both host and parasite fitness, it is important to understand the spatial distribution of coinfections and their drivers. Haemosporidian parasites (Order Apicomplexa) are hyper-diverse and globally distributed (Valkiūnas, 2005; Clark *et al*., 2014), yet few studies have identified the determinants of coinfection within this taxonomic group, and those that have focused on relatively restricted spatial scales. For instance, Oakgrove *et al*. (2014) found an effect of temperature, precipitation and tree cover on blood parasite coinfection rates in birds across several sites in Alaska. Galen *et al*. (2019) also studied avian haemosporidian coinfection in Alaska but focused on the role of evolutionary history in structuring coinfection frequency, finding that distantly related parasites were more likely to occur together within coinfections. Avian haemosporidians are particularly well-suited for studying drivers of coinfections, as birds are commonly infected with haemosporidians in 3 genera – *Haemoproteus* (including *Parahaemoproteus*), *Leucocytozoon* and *Plasmodium* – and haemosporidian coinfection rates exceed 50% of all sampled hosts in some bird populations (van Rooyen *et al*., 2013; Galen *et al*., 2019).

Another underexplored facet of haemosporidian coinfection is the effect of species identity on the frequency and composition of coinfection. Closely related host species that exist across parapatric boundaries are useful for this purpose, as it is possible to assess how the dynamics of coinfection change (or do not) from 1 species to the next despite relatively small geographic distances. There is evidence to suggest that parasites could help maintain parapatric boundaries among species if there is differential susceptibility to the co-evolved parasites of each host species (Thornhill and Fincher, 2013; Theodosopoulos *et al*., 2019), or increased susceptibility of hybrid offspring to parasite infection (Sage *et al*., 1986; Wolinska *et al*., 2004). If coinfections have greater virulence and fitness costs than single infections, a higher prevalence of coinfections could accentuate this ‘wedge effect’ and help to maintain species boundaries. However, in general studies of parasite abundance and diversity across parapatric boundaries are scarce. Reullier *et al*. (2006) noted that sister species of *Hippolais* warbler across a parapatric boundary in Europe harboured different blood parasite assemblages, but coinfections were not reported. Rice *et al*. (2021) studied blood parasites across a hybrid zone of 2 parapatric chickadee species but found a low rate of coinfection. Coinfections have yet to be studied across a parapatric range boundary of a system in which coinfections are common.

**Fig. 1.**
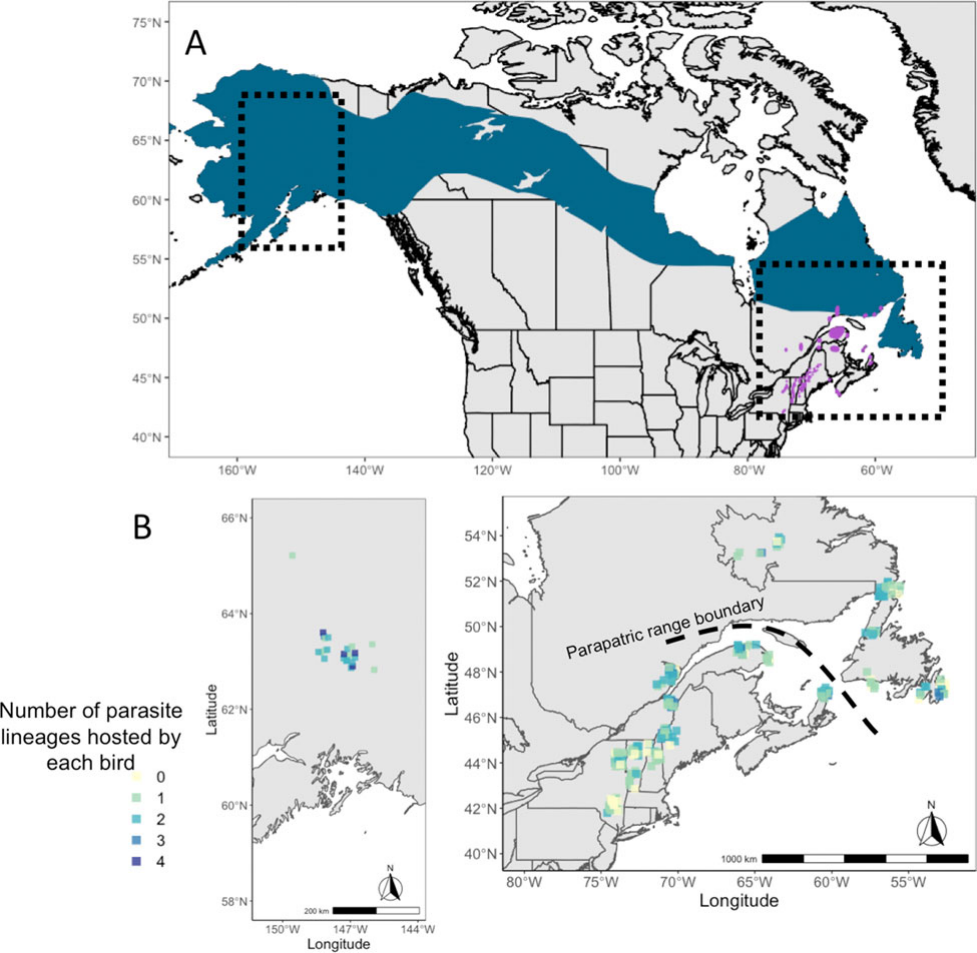
(A) Map of breeding ranges of host species: grey-cheeked thrush (blue) and Bicknell's thrush (purple). Shape files were provided by Birdlife International and Handbook of Birds of the World (2021). (B) Maps showing the localities of sampling of both host species and the number of infections identified in individual birds (ranging from 0 to 4 infections).

The Bicknell's thrush (*Catharus bicknelli*) and grey-cheeked thrush (*Catharus minimus*) are sister species of migratory songbirds that occupy parapatric breeding ranges in North America (Fig. 1) and were likely more geographically disparate during the Pleistocene (FitzGerald *et al*., 2020). These species were elevated from sub-species to full species due to recognition of significant differences in morphology, vocalizations and habitats (Ouellet, 1993), and confirmed genetically more recently (FitzGerald *et al*., 2020; Termignoni-Garcia *et al*., 2022). These 2 species are similar in their high rates and diversity of haemosporidian infections, with especially high prevalence of parasites in the understudied genus *Leucocytozoon* (Starkloff *et al*., 2020). While the occurrence of haemosporidian coinfections has been documented in both the Bicknell's thrush (Starkloff *et al*., 2021) and grey-cheeked thrush (Galen *et al*., 2019), their patterns and drivers are yet to be studied in detail. The Bicknell's thrush has been listed as vulnerable in The International Union for Conservation of Nature's (IUCN) RedList due to population threats such as habitat loss and climate change (IUCN, 2022). Haemosporidians have high endemic prevalence in the Bicknell's thrush, reaching up to 100% in several sites (Starkloff *et al*., 2020). If coinfections do, indeed, lead to higher virulence than single infections, coinfections may pose an added threat to population persistence.

In this study, we first document the variability in rates of haemosporidian infection and coinfections across the parapatric ranges of sister species of thrushes. We then ask the following question: does host species identity, the abiotic environment or latitude best explain variation in haemosporidian coinfection prevalence and patterns of parasite co-occurrence within individual hosts? We use generalized linear mixed-effects models to predict variation in haemosporidian infection and coinfection, as well as a Markov random fields approach (MRFcov; Clark *et al*., 2018) to evaluate specific patterns of haemosporidian lineage co-occurrence within hosts and quantify if abiotic or biotic factors underlie these associations.

## Methods and materials

### Molecular screening and sequencing of haemosporidians

We studied blood and tissue samples collected from Bicknell's thrushes (*n* = 207) and grey-cheeked thrushes (*n* = 91) that are archived in the tissue collections of the American Museum of Natural History and the New York State Museum (see Supplementary Table 1 for specimen numbers). These samples were originally collected from adult birds captured during the breeding season between 1993 and 2018 across 13 sites in eastern and western North America (Fig. 1; Galen *et al*., 2019; Starkloff *et al*., 2020).

Genomic DNA was extracted from each sample using DNeasy Blood and Tissue kits (Qiagen, Valencia, CA, USA). We used a nested polymerase chain reaction (PCR) approach to amplify haemosporidian cytochrome-*b* lineages in genera *Plasmodium*, *Haemoproteus* and *Leucocytozoon* described in Hellgren *et al*. (2004). Positive amplicons were sent for Sanger sequencing on an ABI13700 and sequences were aligned and edited in Geneious Prime (version 2019.1.1). Lineages were identified by matching sequences to known haemosporidian lineages in the MalAvi database (Bensch *et al*., 2009). All lineages that did not 100% match known lineages were provided with a new name according to MalAvi guidelines (CATBIC10-13 and CATMIN11).

### Identification of coinfections

Coinfections within an individual bird were identified by the presence of overlapping peaks in chromatograms. When these peaks were clearly visible in both directions of the reads that composed of an amplicon and the peaks were of different heights (suggesting that 1 infection was dominant), we deciphered the coinfected lineages by manually phasing the sequences (Galen *et al*., 2019). Briefly, the sequence of the dominant infection was subjected to the BLAST function within MalAvi and the best match was aligned to the original contig in Geneious Prime. We were then able to manually identify the sequence of the infection that produced the lower peaks on the chromatogram, which was also subjected to MalAvi BLAST and identified. Each author independently carried out the manual phasing process of each co-infected contig and the identities of co-infecting lineages were only finalized through agreement by both authors. If specific secondary lineages were not clearly decipherable and could not be agreed on by both authors, we noted the additional unknown infection but did not attempt to assign a lineage name.

### Generalized mixed-effects models (GLMMs)

We utilized individual binomial infection and coinfection data within generalized linear mixed-effects models (R package glmmTMB, family ‘binomial’) to test the capacity of host species, latitude and year to predict the variability in haemosporidian infection and coinfection prevalence within host populations. Separate models were run for infection prevalence of each of the 3 haemosporidian genera, for coinfection prevalence of all genera combined and coinfection prevalence of only *Leucocytozoon* parasites. We evaluated the predictors of *Leucocytozoon* coinfection due to its high occurrence in *Catharus* thrushes (Starkloff *et al*., 2020).

Host species was included as a categorical fixed effect to assess if infection and coinfection prevalence vary between hosts. We included latitude as a continuous predictive fixed effect as the studied host species replace one another latitudinally across North America and previous studies have documented turnover in haemosporidian communities with latitude (Starkloff *et al*., 2020). Due to the variability in sampling timespan across sites (ranging from 1 to 21 years), we included a fixed effect of sampling year centred relative to the site mean, a random slope of this site-centred sampling year and random intercept of site. However, we excluded the random slope in the 3 infection prevalence models, as model convergence was challenged by a consistent estimate of variance ≈0. All models were rerun excluding west coast sampling (Alaska, *n* = 24), to check if results were consistent with the exclusion of a single geographically disparate site.

### Markov random fields models

We used the R package MRFcov (Clark *et al*., 2018) to model the co-occurrence of haemosporidians within infected avian host individuals using conditional random fields (CRFs). CRFs use environmental covariates and the existence of other species (in this case other haemosporidian lineages) as predictors using separate regressions to model the occurrence of a species within a network. Here, we considered the ‘sites’ within the network to be *Catharus* host individuals. The method produces estimates of the effect sizes of biotic associations and environmental covariates on the occurrence of each species and can identify estimates of the strength of species associations. As this analysis requires modest sample sizes to model species co-occurrences, we removed all lineages with fewer than 10 total detections in our dataset.

We included several abiotic and biotic environmental covariates in the model. We extracted the values of 19 Bioclim variables (Fick and Hijmans, 2017) for the locality (latitude and longitude) of each individual bird. Thereafter, we performed a redundancy analysis (*RDA* function in R package ‘vegan’; Oksanen *et al.*, 2022) to create 2 variables to use as our abiotic predictors. The top loadings for PC1 were temperature seasonality (negative), annual precipitation (positive) and precipitation in the coldest quarter (positive). For PC2, the top loadings were annual precipitation, precipitation in the driest quarter and precipitation in the warmest quarter (all negative). We also used modelled geographic distribution layers of 4 tree species of importance in our focal avian species (FitzGerald, 2017): paper birch (*Betula papyrifera*), balsam fir (*Abies balsamea*), black spruce (*Picea mariana*) and white spruce (*Picea glauca*). Values of tree species relative to abundance were extracted following the methods described in FitzGerald (2017) for the locality of each individual bird. All environmental covariates used in the model were scaled using the *scale* function in R. Finally, we used host species as a categorical covariate in the model. We ran a spatial model that incorporated the latitude and longitude of each parasite occurrence, and parasite occurrence in each host was treated as a binomial (1 = present, 0 = absent).

## Results

### Haemosporidian distribution, diversity and coinfection rates across sites

Haemosporidians were abundant and diverse in both species of *Catharus*. Across both host species, 86% of all individuals were infected by at least 1 haemosporidian, with the rate of infection in Bicknell's thrush (87%) not different from grey-cheeked thrushes (82%; *χ*^2^ = 0.72, *df* = 1, *P* = 0.40). We detected 34 total haemosporidian lineages (25 *Leucocytozoon*, 3 *Haemoproteus* and 6 *Plasmodium*), with 28 lineages found infecting Bicknell's thrushes, 18 infecting grey-cheeked thrushes and 12 lineages shared between the 2 parapatric host species. Several noteworthy qualitative patterns of lineage occurrence were observed. Three lineages (L_CATGUT02, L_CATMIN04 and L_CATMIN06) were found only in the Alaska sampling sites that were geographically distant from the sites in the northeast. Lineage L_CATGUT02 differs from L_CATMIN07 by only 3 base pairs, but the 2 lineages had non-overlapping distributions with L_CATMIN07 prevalent in both host species (28% of hosts infected) in eastern North America, and L_CATGUT02 was abundant (50% prevalence) in our Alaska sites where only grey-cheeked thrush occurs. Lineage L_CATUST09 was found in grey-cheeked thrush populations in both Alaska and the Northeast, but was found only in the northernmost populations of the Bicknell's thrush. No haemosporidian lineage from the eastern sites (i.e. excluding Alaska) that was found more than twice was exclusive to either host species.

The 34 detected haemosporidian lineages exhibited widely divergent abundances, with 1 lineage (L_CATUST11) making up 30% of all parasite detections (122 detections of L_CATUST11 out of 405 total parasites). However, most lineages were rare with 20 lineages detected just 1 or 2 times. We observed a tendency for rare lineages to be weakly diverged from a more common lineage, as 17 of the rare lineages were 1–2 base pairs diverged from a lineage with at least 12 detections (Supplementary Fig. 1). Sixteen of the rare lineages that were also weakly diverged from a more common lineage were in the genus *Leucocytozoon*, with one of them being in the genus *Haemoproteus*.

Co-occurrence of haemosporidian lineages within a single host individual was common in both species of *Catharus* thrushes, with high variability in coinfection rates ranging from 7 to 75% of all hosts across sites (Table 1). Coinfection of multiple *Leucocytozoon* lineages was most common; 24% of all Bicknell's thrushes and 31% of all grey-cheeked thrushes harboured *Leucocytozoon* coinfections in this study (Table 1). The number of haemosporidian infections found within a single *Catharus* thrush ranged from 1 to 4, with an average of 1.36 infections per sampled host (Fig. 1).

**Table 1.** Haemosporidian coinfection rates across sites

Region	Host	Mean latitude	Haemosporidian coinfection rate (all genera)	*Leucocytozoon* coinfection rate
Catskills (New York)	*bicknelli* (*n* = 41)	42.16	0.07	0
Greens (Vermont and New Hampshire)	*bicknelli* (*n* = 27)	44.11	0.22	0.04
Adirondacks (New York)	*bicknelli* (*n* = 25)	44.19	0.20	0.04
Whites (New Hampshire and Maine)	*bicknelli* (*n* = 20)	44.49	0.25	0.10
Mont Gosford	*bicknelli* (*n* = 20)	45.3	0.75	0.6
Massif du Sud	*bicknelli* (*n* = 19)	46.61	0.7	0.45
Nova Scotia	*bicknelli* (*n* = 15)	47.06	0.6	0.47
Lac Poulin	*bicknelli* (*n* = 20)	47.91	0.65	0.4
Gaspesie	*bicknelli* (*n* = 20)	48.87	0.45	0.45
	Bicknell's thrush (total, *n* = 207)		0.38	0.24
South Newfoundland	*minimus* (*n* = 29)	47.34	0.24	0.14
North Newfoundland	*minimus* (*n* = 15)	50.18	0.4	0
Labrador	*minimus* (*n* = 23)	52.51	0.65	0.35
Alaska	*minimus* (*n* = 24)	63.24	0.71	0.67
	Grey-cheeked thrush (total, *n* = 91)		0.49	0.31

Host species sampled at each location, the mean latitude for all sampling sites within a region, the haemosporidian coinfection rate at each location when considering all haemosporidians and the coinfection rate at each location for only *Leucocytozoon* are shown.

### Generalized linear mixed-effects models

**Fig. 2.**
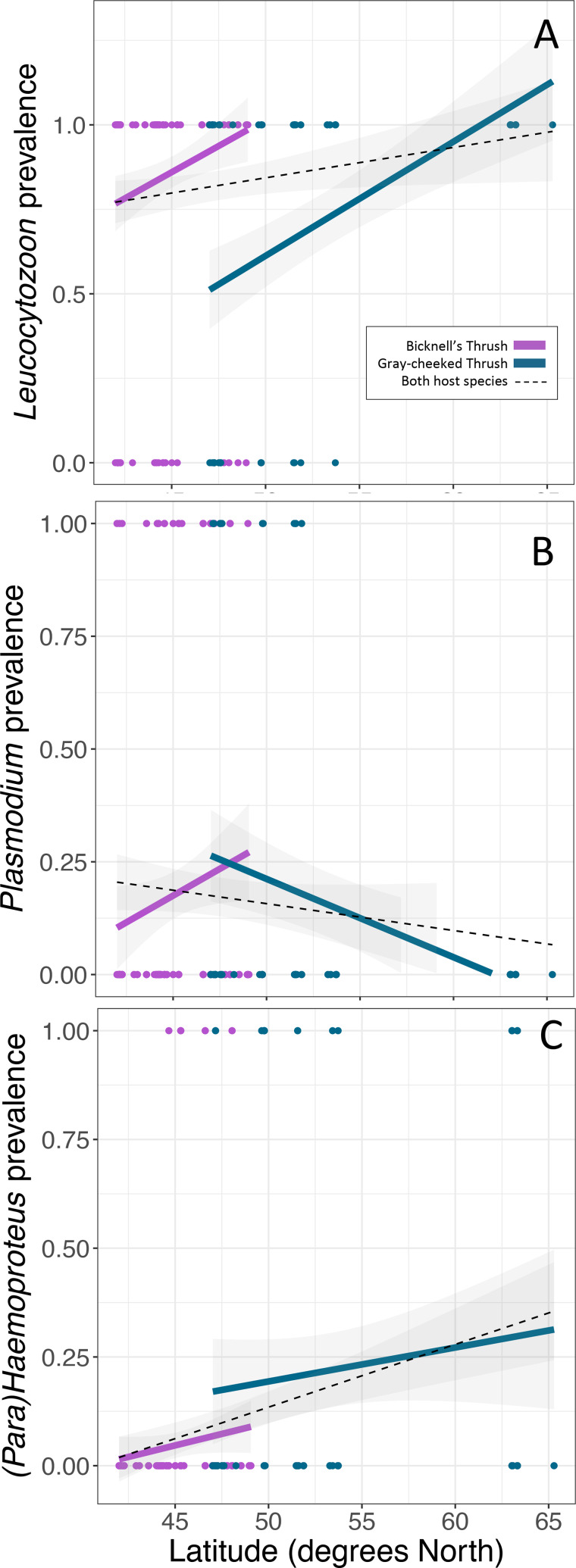
Variability in prevalence of haemosporidians with latitude of (A) *Leucocytozoon*, (B) *Plasmodium* and (C) *Haemoproteus*. Linear regressions are fitted for each host species (same colours as Fig. 1) and for both host species combined (black-dotted line). Points indicate the infection status of individual hosts of each species.

**Fig. 3.**
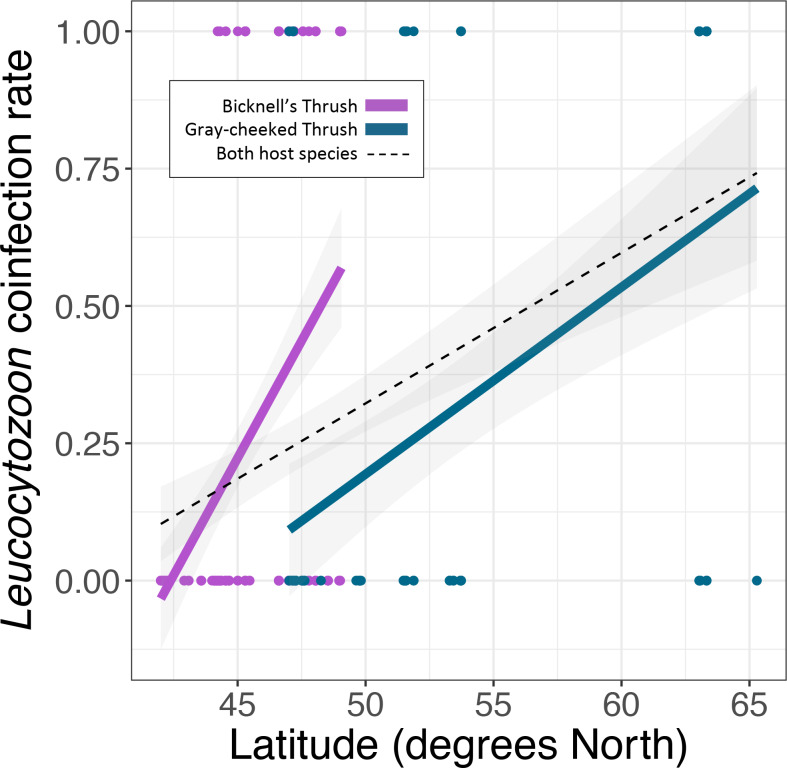
Variability in *Leucocytozoon* coinfection probability with latitude. Linear regressions are fitted for each host species (same colours as Fig. 1) and for both host species combined (black-dotted line). Points indicate the infection status of individual hosts of each species.

*Leucocytozoon* infections were documented in the majority of birds in this study, with higher infection rates in the Bicknell's thrush compared to the grey-cheeked thrush (*P* < 0.001, Table 2, Fig. 2A), though *Leucocytozoon* coinfection rates did not vary between the 2 species (*P* = 0.068, Table 2, Fig. 3). Both *Leucocytozoon* infection and coinfection were positively correlated with latitude; *P* < 0.001 (Fig. 2A) and *P* = 0.003 (Fig. 3), respectively. Coinfections including all 3 genera (Table 2) also increased with latitude (*P* = 0.039) and did not differ between host species (*P* = 0.624). Variation in *Plasmodium* and *Haemoproteus* prevalence (Fig. 2B and C) was not well predicted by any of the 3 fixed effects included in the model (Table 2); however, due to the diametric *Plasmodium* infection patterns on the 2 host species (Fig. 2B), we repeated the *Plasmodium* model with an interaction term between host species and latitude. This led to a stronger model fit (ΔAIC of 7.6 and interaction term *P* = 0.005), suggesting that the 2 host species have largely deviating slopes. Models run with data that excluded sampling from Alaska yielded similar outcomes, with the exception of host species being a significant predictor of *Leucocytozoon* coinfection (*P* = 0.018, Supplementary Table 2).

**Table 2.** Model output of 5 generalized linear mixed-effects models predicting prevalence of separate haemosporidian genera and coinfections in parapatric host thrushes (*n* = 298)

Dependent variable	Independent variable	Estimate	*P* value	Standard error
*Leucocytozoon* Prevalence	HostSpecies	−3.08758 (relative to *Catharus bicknelli*)	**<0.001**	0.53364
	Latitude	0.35722	**<0**.**001**	0.07830
	Year (site-centred)	−0.02813	0.308	0.02762
*Plasmodium* Prevalence	HostSpecies	0.37672 (relative to *C. bicknelli*)	0.563	0.65158
	Latitude	−0.10344	0.164	0.07429
	Year (site-centred)	0.05861	0.059	0.03105
*Haemoproteus* Prevalence	HostSpecies	1.65742 (relative to *C. bicknelli*)	0.116	1.05404
	Latitude	0.04796	0.570	0.08433
	Year (site-centred)	0.04223	0.406	0.05084
Haemosporidian coinfection rate (all genera)	HostSpecies	−0.44593 (relative to *C. bicknelli*)	0.624	0.90971
	Latitude	0.16296	**0**.**039**	0.07874
	Year (site-centred)	0.08307	0.237	0.07028
*Leucocytozoon* coinfection rate	HostSpecies	−2.19556 (relative to *C. bicknelli*)	0.068	1.20477
	Latitude	0.28209	**0**.**004**	0.09690
	Year (site-centred)	0.10233	0.192	0.07851

Three predictive variables were included in all models (host species, latitude and sampling year). All models included a random intercept of sampling site and the 2 coinfection models included site-centred year as a random slope. P values bolded when <0.05.

While climatic variables were considered, they were not included in GLMMs due to multi-collinearity with latitude (correlation coefficients of 0.52 and −0.74 for PC1 and PC2, respectively). However, if we consider the top loadings of PC1 and PC2, increases in *Leucocytozoon* infection and coinfection prevalence with latitude also correlate with increases in annual precipitation and in precipitation in the driest, warmest and coldest quarters as well as with decreases in temperature seasonality.

### MRFcov analysis

**Fig. 4.**
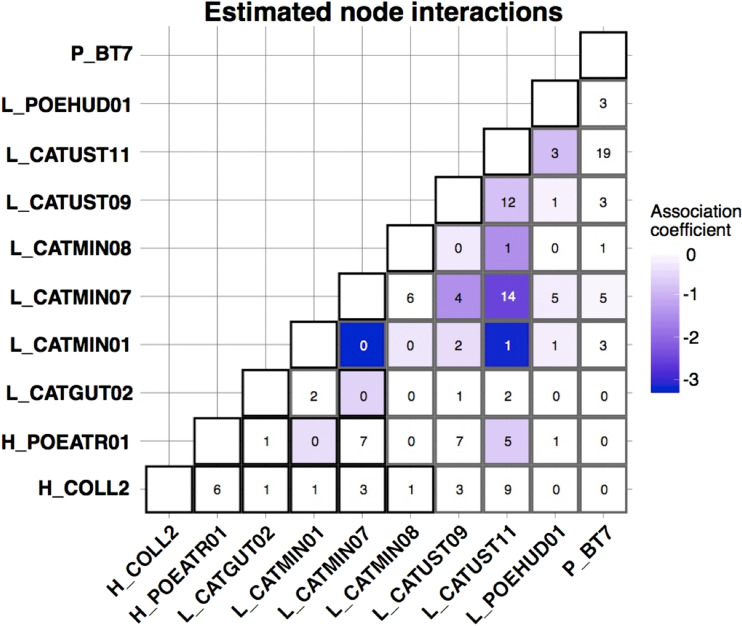
Coefficient outputs of the CRF model produced by MRFcov. Each cell of the matrix contains the number of co-occurrences of 2 haemosporidian lineages that we found across all host species, with the colour of the cell shown based on the estimated association coefficient with darker colours indicating a more negative association coefficient.

The CRF model run using MRFcov revealed associations among haemosporidian lineages with coefficients that were approximately 0 or were negative, indicating that these lineages tended to occur together in coinfections less often than expected (Fig. 4). The 2 strongest negative associations involved lineage L_CATMIN01 (17 total infections across all hosts): L_CATMIN07 (78 total infections across all hosts) never occurred in a coinfection with L_CATMIN01, and L_CATUST11 (122 total infections) occurred in just 1 coinfection with L_CATMIN01.

In nearly every instance, the most important variable for the occurrence of each haemosporidian lineage was the occurrence of a different haemosporidian, not an environmental or host covariate (Supplementary Table 3). The one exception was for the occurrence of *Haemoproteus* lineage H_COLL2, for which the most important covariate was host species. H_COLL2 was found predominantly in grey-cheeked thrush (13 infections), with Bicknell's thrush being a rare host for this parasite (3 infections).

## Discussion

Latitudinal diversity gradients, typically characterized by increasing diversity towards lower latitudes, are common in many free-living taxa (Hillebrand, 2004) but are not well examined in parasitic taxa. Several studies of parasitic taxa have instead revealed gradients of parasite diversity or abundance which increase towards higher latitudes (Cuevas *et al*., 2020; Fecchio *et al*., 2020; Johnson and Haas, 2021). We similarly found an increase in the probability of haemosporidian infection with increasing latitudes in our parapatric host system, though to our knowledge our study is the first to find evidence for a latitudinal gradient of coinfections in a eukaryotic parasite system. A single study documented a comparable finding in grassland viral pathogens, though using a different metric: the number of viruses infecting a single host increased 2-fold across their western North American gradient (Seabloom *et al*., 2010). We found a similar pattern of increasing number of infections per host with latitude in our system (Fig. 1B).

The latitudinal pattern that we observed was driven heavily by the haemosporidian genus *Leucocytozoon*, which are vectored by black flies in the family Simuliidae and have been shown to be more diverse and prevalent at higher latitudes (Fecchio *et al*., 2020). Understanding this parasite's biogeographic pattern relies on an understanding of the black fly vector and far less is known about how the diversity and abundance of this Dipteran group changes with latitude. Black fly species richness does appear to increase towards higher latitudes in North America, with an estimated peak black fly richness between 50 and 53° latitude followed by a decline towards the highest latitudes (McCreadie *et al*., 2017). Changes in black fly abundance across space are not well-studied; McCreadie and Adler (2014) found an association between latitude and black fly occupancy in South Carolina, USA, though additional work is needed to determine the generality of this pattern at a continental scale. Collectively, the drivers of the reverse latitudinal gradient in coinfection that we observed here deserve future attention, particularly if this pattern proves to be more widespread in other host–parasite systems.

Chronic haemosporidian infections can reduce lifespan and fecundity in birds (Asghar *et al*., 2015), thus the observation that higher latitude thrushes appear to accumulate more infections could have implications for the health of these populations. A higher parasite burden, in combination with longer migration distances, could act synergistically to reduce lifespan and reproductive success, known as ‘migratory culling’ (Altizer *et al*., 2011). However, it is not clear whether all avian haemosporidians have similarly negative impacts on host fitness, particularly in the understudied genus *Leucocytozoon* which is the most prevalent haemoparasite in high-latitude regions of North America (Fecchio *et al*., 2020). The effect of coinfections from a conservation perspective is important to consider with the Bicknell's thrush, which is of conservation concern due to habitat loss and climate change (IUCN RedList, 2020). Yet as the fitness effects of haemosporidians within specific host species are variable and generally poorly characterized, it is possible that the haemosporidians of *Catharus* thrushes are relatively benign. The long-term chronic effects of haemosporidian coinfection deserve future attention in this system as well as in other migratory host species that face high infection burdens.

Due to the chronic nature of haemosporidian infections, it is challenging to decipher if the presence of multiple lineages in each individual is indicative of the concurrent presence of multiple infections or the history of infections over a bird's lifetime (Valkiūnas, 2005). PCR-based methods can only provide information on the presence of haemosporidian DNA and not if the infection is acute, chronic or may have been abortive and never developed to a transmissible stage (Valkiūnas *et al*., 2008). Further studies using microscopic work would be necessary to confirm active infections, though for parasites such as *Leucocytozoon* it may be impossible to morphologically distinguish among closely related lineages that are involved in coinfections (Galen *et al*., 2018). Regardless of whether these are simultaneously active infections or an indication of haemosporidian infection history, our study suggests that North American thrushes at higher latitudes have higher tendency for infection by a diverse array of blood parasites.

Many of the coinfections that we detected involved multiple, weakly genetically differentiated lineages (often separated by just 1 or 2 nucleotide differences, Supplementary results), and thus may be indistinguishable morphologically. Further investigation is needed to understand the extent to which the coinfections that we documented consisted of multiple species or multiple genotypes of the same species. Species limits have been difficult to diagnose among avian haemosporidians because there is no consistent level of *cytb* divergence that indicates reproductive isolation among different mitochondrial lineages (Bensch *et al*., 2004; Galen *et al*., 2018). A previous study on *Leucocytozoon* species limits by Galen *et al*. (2018) confirmed that several of the common lineages in our study are distinct species, e.g. L_CATUST11 (which was denoted as L_CATMIN05 in that study), L_CATUST09, L_CATMIN01 and L_CATGUT02. However, the species status of many of the remaining 30 lineages that we recovered remains unclear. A systematic effort to investigate nuclear genetic diversity among avian haemosporidian lineages is needed to address this gap in our knowledge.

Parapatric species boundaries may act as a barrier to parasite transmission or provide an opportunity for parasites to expand their geographic ranges (Theodopholous *et al*., 2019). It is challenging to document evidence of these phenomena mechanistically; however, some studies provide documentation of parasite ranges matching host ranges across a parapatric species boundary (Reullier *et al*., 2006). In our study system, host species identity was not a good predictor of coinfection tendency and both sister host species shared the majority of detected haemosporidian lineages. The few lineages that were abundant only in the grey-cheeked thrush were found in the geographically isolated Alaskan site. One lineage of note (L_CATUST09) was found in both host species, but only in the northern sites of the Bicknell's thrush (>45° latitude). This could provide evidence for a historical spillover event from the grey-cheeked thrush which is spreading south into the Bicknell's thrush range, though we acknowledge this interpretation is speculative and that the distribution of L_CATUST09 could be constrained by some other factor such as the presence of a specific vector. One other lineage, H_COLL2, showed a skewed distribution with higher prevalence in grey-cheeked (14.3%) than in Bicknell's thrushes (1.5%), though our sample size was low (16 total infections). Additional work on haemosporidian biogeography or an evaluation of changes in parasite diversity over the next few decades may elucidate if the current parasite distributions are the result of a true spillover event.

Geographic patterns and determinants of haemosporidian coinfection are scarcely studied. We provide evidence of a reverse gradient of coinfection probability in North American thrushes, with higher latitude birds having a higher likelihood of co-occurring infections. There was little evidence for a role of host species in structuring haemosporidian coinfection patterns or coinfection probability in this parapatric host system. Coinfection experiments, evaluation of active infections and vector studies would help decipher the mechanisms behind these propensities for coinfection.

## Data Availability

Haemosporidian DNA sequences (CATBIC10-13 and CATMIN11) are available on the MalAvi database (http://130.235.244.92/Malavi/).

## Supplementary material

For supplementary material accompanying this paper visit https://doi.org/10.1017/S0031182022001792. click here to view supplementary material
