# Supplementary material for: Coinfection rates of avian blood parasites increase with latitude in parapatric host species
Source: Parasitology. 2023 Jan 4;150(4):329–36. doi: 10.1017/S0031182022001792 (PMC10090641; doi:10.1017/S0031182022001792)
Supplement: Supplementary file 1 [file S0031182022001792sup.zip › S0031182022001792sup002.docx]

**Supplementary Figure 1.** Cytochrome b haplotype network produced using the TCS algorithm in PopART of the 34 haemosporidian lineages detected in this study. *Leucocytozoon* lineages are denoted in blue, *Plasmodium* lineages are denoted in red, and *Haemoproteus* lineages are denoted in yellow. Shown in red text and with larger blue circles are *Leucocytozoon* lineages that were particularly abundant in our sample (detected 12 or more times).

**Supplementary Table 2.** Model output of five generalized linear mixed effects models predicting prevalence and coinfections of haemosporidian lineages in parapatric host thrushes excluding west coast sampling (n=274). Three predictive variables were included in the models (host species, latitude, and sampling year centered by site) to evaluate their predictive capacity. All models included a random intercept of sampling site and the coinfection models include site-centered year as a random slope.

| **Dependent Variable** | **Independent variable** | **Estimate** | **P-value** | **Standard error** |
| --- | --- | --- | --- | --- |
| *Leucocytozoon* Prevalence | HostSpecies | -3.04310  (relative to *C. bicknelli*) | **2.39e-08** | 0.54523 |
|  | Latitude | 0.34699 | **2.90e-05** | 0.08299 |
|  | Year (site-centered) | -0.02805 | 0.309657 | 0.02761 |
| *Plasmodium* Prevalence | HostSpecies | -0.05731  (relative to *C. bicknelli*) | 0.917 | 0.55249 |
|  | Latitude | 0.04740 | 0.574 | 0.08428 |
|  | Year (site-centered) | 0.05677 | 0.065 | 0.03077 |
| *(Para) Haemoproteus* Prevalence | HostSpecies | 1.66474  (relative to *C. bicknelli*) | 0.232 | 1.39224 |
|  | Latitude | 0.07199 | 0.727 | 0.20592 |
|  | Year (site-centered) | 0.04816 | 0.361 | 0.05276 |
| Haemosporidian coinfection rate  (all genera) | HostSpecies | -1.14506  (relative to *C. bicknelli*) | 0.0825 | 0.65940 |
|  | Latitude | 0.43272 | **2.70e-06 **** | 0.09222 |
|  | Year | 0.08265 | 0.2676 | 0.07454 |
| *Leucocytozoon* coinfection rate | HostSpecies | -2.6943  (relative to *C. bicknelli*) | **0.018018 *** | 1.1391 |
|  | Latitude | 0.5363 | **0.001634 **** | 0.1703 |
|  | Year (site-centered) | 0.1299 | 0.173842 | 0.0955 |

**Supplementary Table 3.** CRF regression coefficients of haemosporidian lineage infection probability produced by MRFcov. Note that only the ten most abundant lineages were included in this analysis.

| **Parasite lineage** | **Variable** | **Rel_importance** | **Standardised_coef** | **Raw_coef** |
| --- | --- | --- | --- | --- |
| H_COLL2 | Species1 | 1 | 0.497527 | 0.497527 |
| H_POEATR01 | L_CATUST11 | 0.9731689 | -0.7190642 | -0.7190642 |
|  | L_CATMIN01 | 0.0268311 | -0.1193968 | -0.1193968 |
| L_CATGUT02 | L_CATMIN07 | 1 | -0.4595424 | -0.4595424 |
| L_CATMIN01 | L_CATUST11 | 0.5644376 | -2.627631 | -2.627631 |
|  | L_CATMIN07 | 0.4270797 | -2.285656 | -2.285656 |
| L_CATMIN07 | L_CATUST11 | 0.45324400 | -2.4272869 | -2.4272869 |
|  | L_CATMIN01 | 0.40189419 | -2.2856564 | -2.2856564 |
|  | L_CATUST09 | 0.11814765 | -1.2392748 | -1.2392748 |
|  | L_CATGUT02 | 0.01624579 | -0.4595424 | -0.4595424 |
| L_CATMIN08 | L_CATUST11 | 0.90623394 | -1.4548368 | -1.4548368 |
|  | PC1_L_CATUST11 | 0.08004731 | -0.4323819 | -0.4323819 |
|  | L_CATUST09 | 0.01371876 | -0.1789994 | -0.1789994 |
| L_CATUST09 | L_CATMIN07 | 0.56725094 | -1.2392748 | -1.2392748 |
|  | L_CATUST11 | 0.24067416 | -0.8072252 | -0.8072252 |
|  | Abiesbalsamea_L_CATUST11 | 0.15075462 | 0.6388741 | 0.6388741 |
|  | PC1 | 0.02225526 | 0.2454688 | 0.2454688 |
|  | L_CATMIN08 | 0.01183431 | -0.1789994 | -0.1789994 |
| L_CATUST11 | L_CATMIN01 | 0.39111914 | -2.6276314 | -2.6276314 |
|  | L_CATMIN07 | 0.33375086 | -2.4272869 | -2.4272869 |
|  | L_CATMIN08 | 0.11989711 | -1.4548368 | -1.4548368 |
|  | L_POEHUD01 | 0.04595379 | -0.9006801 | -0.9006801 |
|  | L_CATUST09 | 0.03691217 | -0.8072252 | -0.8072252 |
|  | H_POEATR01 | 0.02928974 | -0.7190642 | -0.7190642 |
|  | Abiesbalsamea_L_CATUST09 | 0.02312122 | 0.6388741 | 0.6388741 |
|  | PC1_L_CATMIN08 | 0.01059047 | -0.4323819 | -0.4323819 |
| L_POEHUD01 | L_CATUST11 | 0.92986436 | -0.9006801 | -0.9006801 |
|  | L_CATMIN07 | 0.03774676 | -0.1814682 | -0.1814682 |
|  | L_CATUST09 | 0.01950420 | -0.1304442 | -0.1304442 |
|  | Betulapapyrifera_L_CATMIN07 | 0.01288468 | 0.1060224 | 0.1060224 |
| P_BT7 | L_CATMIN07 | 0.91889723 | -0.071144407 | -0.071144407 |
|  | Abiesbalsamea | 0.04209880 | 0.015227975 | 0.015227975 |
|  | PC2_L_CATMIN07 | 0.02142594 | 0.010863687 | 0.010863687 |
|  | Piceaglauca_L_CATMIN07 | 0.01757803 | -0.009839937 | -0.009839937 |
